# The Intricate Relationship between Psychotic-Like Experiences and Associated Subclinical Symptoms in Healthy Individuals

**DOI:** 10.3389/fpsyg.2017.01537

**Published:** 2017-09-07

**Authors:** Lui Unterrassner, Thomas A. Wyss, Diana Wotruba, Helene Haker, Wulf Rössler

**Affiliations:** ^1^Collegium Helveticum, ETH Zurich and University of Zurich Zurich, Switzerland; ^2^Translational Neuromodeling Unit, Institute for Biomedical Engineering, University of Zurich and ETH Zurich Zurich, Switzerland; ^3^Department of Psychiatry, Psychotherapy and Psychosomatics, Psychiatric Hospital, University of Zurich Zurich, Switzerland; ^4^Department of Psychiatry and Psychotherapy Charité Mitte, Charité, Germany

**Keywords:** psychotic-like experiences, negative symptoms, affective symptoms, depression, anxiety, subclinical psychosis, psychosis continuum, healthy individuals

## Abstract

The interplay between subclinical psychotic, negative, and affective symptoms has gained increased attention regarding the etiology of psychosis spectrum and other mental disorders. Importantly, research has tended to not differentiate between different subtypes of psychotic-like experiences (PLE) although they may not have the same significance for mental health. In order to gain information on the subclinical interplay between specific PLE and other symptoms as well as the significance of PLE for mental health, we investigated their specific associations in 206 healthy individuals (20–60 years, 73 females) using correlational and linear regression analyses. PLE were assessed with the Magical Ideation Questionnaire, the revised Exceptional Experiences Questionnaire, and subscales of the Schizotypal Personality Questionnaire (SPQ). The revised Symptom Checklist 90, the SPQ, and the Physical Anhedonia Scale were used to measure subclinical negative symptoms, affective symptoms, and other symptoms such as, emotional instability. As hypothesized, we found that (1) most affective symptoms and all other subclinical symptoms correlated positively with all PLE, whereas we found only partial associations between negative symptoms and PLE. Notably, (2) magical ideation and paranormal beliefs correlated negatively with physical anhedonia. In the regression analyses we found (3) similar patterns of specific positive associations between PLE and other subclinical symptoms: Suspiciousness was a specific predictor of negative-like symptoms, whereas ideas of reference, unusual perceptual experiences, and dissociative anomalous perceptions specifically predicted anxiety symptoms. Interestingly, (4) ideas of reference negatively predicted physical anhedonia. Similarly, paranormal beliefs were negatively associated with constricted affect. Moreover, odd beliefs were a negative predictor of depression, emotional instability, and unspecific symptoms. Our findings indicated that subtypes of PLE are differentially implicated in psychological functioning and should therefore not be categorized homogeneously. Moreover, paranormal beliefs, odd beliefs, and partly ideas of reference might also contribute to subjective well being in healthy individuals. Our results might serve as a starting point for longitudinal studies investigating the interplay of subtypes of subclinical symptoms along a psychopathological trajectory leading to mental disorders. Importantly, this research might help to improve therapeutic strategies for psychosis prevention.

## Introduction

Psychotic disorders are primarily characterized by the presence of delusions and hallucinations. More recently, awareness has increased that delusions and hallucinations are not only a feature of psychotic disorders but are also subtly present in affective disorders, i.e., depression and anxiety (Olfson et al., 2002; Hanssen et al., 2003; Kelleher and Cannon, 2011; Wigman et al., 2012; Jeppesen et al., 2015). At the same time, symptoms of depression and anxiety are also present in the majority of schizophrenic patients (Huppert and Smith, 2005; Buckley et al., 2009). Hence, the various disorders that belong to the psychotic disorder spectrum might be conceptualized as different manifestations of one syndrome that share etiology and psychopathology (van Os, 2015). For example, schizophrenia represents a syndrome that is characterized by long duration, delusions, negative symptoms (reduction in drive and volition), and a few affective symptoms. In contrast, patients with fewer negative and psychotic symptoms but with a high prevalence of affective symptoms might be diagnosed with bipolar disorder (van Os and Kapur, 2009). Evidence suggests that fundamental trans-diagnostic associations between different psychopathological domains such as, psychotic symptoms, affective symptoms, negative symptoms, and disorganized symptoms (difficulties in memory, attention, and executive functions) extend from subclinical to clinical symptom levels (van Os and Reininghaus, 2016).

In their seminal longitudinal study, Chapman et al. (1994) demonstrated that mild and/or transient forms of psychotic experiences reported by individuals with a schizophrenia diagnosis confer a higher risk for psychosis in healthy individuals. Importantly, when negative symptoms were included as predictors, the rate of transition to psychosis increased markedly (Chapman et al., 1994; Kwapil, 1998; Gooding et al., 2005). These studies suggested that investigating early psychotic symptoms and negative-like symptoms might specifically inform on the development of later psychotic disorders. However, more recently it has been suggested that a transdiagnostic approach to investigating psychosis might be more appropriate, as symptoms are not restricted to diagnostic boundaries and might cross symptom domains in their development (Wigman et al., 2017). For example, other symptoms such as, depression might also predict later psychotic disorder (Yung et al., 2004) and psychotic symptoms in turn, might also predict nonpsychotic disorders (Fusar-Poli et al., 2012; Lin et al., 2015). Therefore, studies investigating the etiology of psychotic disorders should include experiences from multiple domains of psychopathology, such as, positive psychotic experiences, negative psychotic experiences, anxiety, and depression. More recently, it has been proposed that in pre-morbid stages of mental disorders symptoms are rather unspecific and may drive the development of distinct disorders through mutually affecting interplays (Borsboom and Cramer, 2013; Wigman et al., 2017). In order to pinpoint mechanisms between subclinical psychotic and co-morbid symptoms in the pathogenesis of psychosis, the initial, or subclinical levels of this interplay should be investigated in particular (Rössler et al., 2011).

A central question in research focusing on subclinical psychotic symptoms or psychotic-like experiences (PLE) is, whether they equally affect functioning and well-being. Yung and Lin (2016) suggested that the type of PLE may even play a role in determining if an individual develops psychotic disorder or more common mental disorders. van Os and Reininghaus (2016) and Yung et al. (2009) have proposed similar models, suggesting that subtypes of PLE can be categorized into three basic categories. Some might indicate a specific vulnerability toward psychosis (category 1), whereas others might be non-specific and also be implicated in the development of affective disorders (category 2) or might not be associated with any clinical disorder at all (category 3). Yung and Lin (2016) proposed that PLE that are associated with depression, distress, and poor functioning might particularly be indicative of vulnerability toward psychosis. At the same time, features such as, magical thinking might not necessarily be maladaptive (Yung et al., 2006). However, in the literature on PLE, there has been a tendency not to differentiate between different types of experiences (Yung et al., 2009).

Recently, we investigated PLE in a sample of healthy individuals from the general population (Unterrassner et al., 2017) using the Schizotypal Personality Questionnaire (SPQ; Raine, 1991), the revised Symptom Checklist 90 (SCL-90-R; Derogatis, 1977; Rössler et al., 2007), and the recently developed PAGE-R questionnaire (Fach et al., 2013; Landolt et al., 2014). Based on our results, we suggested that the association of psychotic experiences with socio-demographic factors and reduced psychological functioning (comorbid symptoms, psychological distress, lower educational achievement) extends to the healthy end of the psychosis continuum (Linscott and van Os, 2013), i.e., healthy individuals from the general population. Moreover, we identified three factors underlying the experiences in the PAGE-R: (1) Odd beliefs, comprising experiences such as, seeing meaning in coincidences or the anticipation of future events, (2) dissociative anomalous perceptions, encompassing for example the autonomous activity of body parts or the alienation to one's own personality, and (3) hallucinatory anomalous perceptions, referring to experiences such as, hearing inexplicable noises or different hypnagogic perceptions. Importantly, these three subtypes of PLE were differentially associated with distress and comfort. Whereas hallucinatory anomalous perceptions were particularly burdensome, odd beliefs were foremost comforting. Furthermore, we found that physical anhedonia was negatively correlated with paranormal beliefs SPQ and odd beliefs (PAGE-R; trend). These results were in line with earlier “paradoxical” findings showing negative associations between magical ideation and physical anhedonia (see Chapman et al., 1982). These negative associations indicated that subtypes of PLE might differently affect mental health and that it might be important to differentiate between them (Brett et al., 2014; Yung and Lin, 2016). For example, it is conceivable that odd beliefs reduce physical anhedonia by conveying meaning to experiences.

In this study, we aimed at gaining a deeper understanding of the relationships between subtypes of PLE and associated “co-morbid” symptoms in healthy individuals, such as, anxiety symptoms. We sought to extend our previous findings using a sample of healthy individuals from the general population (Unterrassner et al., 2017). We investigated specific associations between subtypes of PLE and three groups of symptoms: (1) negative-like symptoms, (2) affective symptoms, and (3) other subclinical symptoms that may be present in psychiatrically healthy individuals from the general population, such as, emotional instability. PLE were assessed using the Magical Ideation Scale (Eckblad and Chapman, 1983), the psychotic-like SPQ subscales (Adrian Raine et al., 1994), and the PAGE-R subscales odd beliefs, dissociative anomalous perceptions, and hallucinatory anomalous perceptions (Unterrassner et al., 2017). The Physical Anhedonia Scale (PAS; Chapman et al., 1976), the no close friends subscale as well as the constricted affect subscale of the SPQ were used to assess negative-like symptoms. Affective symptoms were measured using the SCL-90-R subscales depression, anxiety, phobic anxiety, obsessive-compulsive, somatization, and the SPQ subscale social anxiety. Other subclinical symptoms implicated in well-being encompassed the SCL-90-R subscales interpersonal sensitivity, anger-hostility, and unspecific symptoms.

To the best of our knowledge this is the first study to investigate specific associations between PLE and negative-like symptoms, affective symptoms, and other subclinical symptoms in healthy individuals using correlational and regression analyses. In extension of our earlier findings (Unterrassner et al., 2017), we hypothesized (1) PLE to be positively correlated with negative-like symptoms, affective symptoms, and other subclinical symptoms. However, based on the results by Chapman et al. (1982), we hypothesized that (2) magical ideation, paranormal beliefs, and odd beliefs are negatively correlated with physical anhedonia. In addition to testing the correlational associations between subtypes of PLE and other subclinical symptoms, we aimed at identifying unique associations between subtypes of PLE and negative-like symptoms, affective symptoms, and other subclinical symptoms using hierarchical multiple linear regression modeling. Based on the findings by Yung et al. (2006) we hypothesized that (3) magical ideation, paranormal beliefs, and odd beliefs negatively predict depressive symptoms.

Studying the interrelations between subclinical symptoms in healthy individuals might point toward cognitive mechanisms that are involved in the maintenance of healthy psychological functioning or the exacerbation of psychopathological symptoms, respectively. Importantly, the identification of such mechanisms could help to develop strategies in psychosis prevention.

## Materials and methods

### Study design and sampling

In 2011, PLE and help-seeking behavior were assessed in a large online sample representative of the Swiss general population (*N* = 1,580; Fach et al., 2013; Landolt et al., 2014). Ninety-one individuals from the aforementioned survey agreed to take part in this follow up study. One hundred and forty-six additional participants from the general population were subsequently acquired by online ads, pamphlets, and word-of-mouth. This analysis is based on level 1 data of the follow-up study of the “Exceptional Experiences” project (please see Unterrassner et al., 2017, for a study overview).

### Inclusion/exclusion criteria

Eligible subjects were between 20 and 60 years of age and in good command of the German language. Several self-reported confounding factors resulted in the exclusion of 18 individuals (7.6%) from the analysis: A parent with history of psychosis (1), multiple sclerosis (1), Guillain-Barré-Syndrome (1), attention deficit hyperactivity disorder (3), craniocerebral trauma (1), current use of antidepressants (1), consumption of drugs other than alcohol, nicotine or cannabis >10 days before the examination or on a regular basis (3), for practical reasons nicotine craving within 4 h of deprivation (2), insufficient German skills (1), epilepsy (2), panic attacks (1), and episodes of bodily numbing (1). The “caseness” criteria for psychoticism and paranoia as operationalized by the SCL-90-R definition (Derogatis, 1983) was applied in order to control for potential psychopathology, which led to the exclusion of 13 individuals from the analysis. Seventy-three women and 133 men (*M*_SPQ_ = 18.86, *SD* = 10.56, Range = 0–66) were included in the final analysis (*M*_age_ = 33.11 years, *SD* = 11.23, Range = 20–60).

The study was conducted in accordance with principles enunciated in the “Declaration of Helsinki,” the guidelines of Good Clinical Practice (GCP) and Swiss regulatory authority's requirements. Volunteers gave fully written consent for the study, which was approved by the sub-commission of the ethics committee of the Canton of Zurich, specialized on psychiatry, neurology, neurosurgery, and health care science (Department D; Prof. Dr. med. Dr. phil. Paul Hoff; KEK-ZH, Nr. 2011-0423).

### Measures

#### The SCL-90-R

The Symptom Checklist-90-Revised (SCL-90-R; Derogatis, 1977) inquires on general and particular psychopathology in the last few weeks on five-point Likert-scales, ranging from 0 (*not at all*) to 4 (*extremely*). The paranoia subscale and the psychoticism subscale were used to control the sample for potential psychotic cases. General psychological distress was operationalized with the global severity index (GSI) that reflects the mean psychological distress across all 90 symptoms. For the present study, we used the SCL-90-R subscales assessing affective symptoms (anxiety, phobic anxiety, obsessive-compulsive, somatization, depression) and other subclinical symptoms (interpersonal sensitivity, anger-hostility, additional items). The anger-hostility subscale measures irritability and emotional instability through strong aggressiveness. Notably, this subscale might partly depict the personality trait emotional instability, which encompasses anxiety, moodiness, low resiliency, or jealousy (Thompson, 2008). Therefore, this subscale is referred to as emotional instability (anger-hostility) hereafter. The additional items subscale assesses unspecific disturbances in eating and sleeping behavior and will be referred to as “unspecific symptoms.” We applied the “caseness criterion” to exclude individuals expressing symptoms that might be of clinical relevance (Derogatis, 1977). Thirteen subjects that were above a cut-off of 63 in both the t-transformed paranoia subscale and the psychoticism subscale were removed from the analysis.

#### The SPQ

The SPQ comprises 74 forced-choice items (*yes* or *no*) distributed over nine subscales (Raine, 1991). We assessed PLE using following subscales: Ideas of reference, paranormal beliefs, unusual perceptual experiences, and suspiciousness (Raine et al., 1994). The SPQ subscale, no close friends, was used to assess a facet of negative-like symptoms, i.e., a disinterest in social interactions. The social anxiety subscale was used as a measure for anxiety in addition to the aforementioned SCL-90-R subscales anxiety, phobic anxiety, obsessive-compulsive, and somatization.

#### The PAGE-R

The Revised Exceptional Experiences Questionnaire (PAGER; Fach et al., 2013; Landolt et al., 2014) assesses the frequency of 32 exceptional experiences on five-point Likert-scales ranging from 0 (*never*) to 4 (*very often*). We implemented the PAGE-R as a measure for PLE using three subscales (Unterrassner et al., 2017): The odd beliefs subscale (OB), the dissociative anomalous perceptions subscale (DAP), and the hallucinatory anomalous perceptions subscale (HAP). The questionnaire concludes with information on socioeconomic variables, including age, sex, and educational achievement. Educational achievement was indicated on a five-point Likert-scale (0 = *no education*, 1 = *compulsory school*, 2 = *vocational training*, 3 = *high-school diploma*, 4 = *university or another type of higher education*).

#### The PAS

The PAS (Chapman et al., 1976) comprises 30 dichotomous items (*yes* or *no*) that assess the inability to enjoy sensory stimuli such as, eating, feeling, sex, smell, and sound. The PAS served as a measure for negative-like symptoms.

#### The MIS

The Magical Ideation Scale (MIS; Eckblad and Chapman, 1983) makes various statements of belief and experiences with which participants can either agree or disagree (*yes* or *no*). Notably, the MIS assesses PLE but comprises a number of different constructs rather than a single one and includes for example, paranormal beliefs, superstitious beliefs, ideas of reference, and suspicious-paranoid thoughts (Vyse, 1997). For this reason, the MIS was only included for the correlational analyses but was not included as a predictor in the hierarchical multiple linear regression models.

### Statistical analysis

#### Missing data replacement

The expectation maximization algorithm in the SPSS 21 missing value analysis procedure was used to replace missing data in the PAGE-R and SCL-90-R datasets (IBM Corporation, 2012). The PAGE-R data was lacking five data points (0.07%) and the SCL-90-R data 69 data points (0.32%; see Supplementary Table 1). The PAS data was missing 3 cases.

#### Spearman's correlations

PLE as operationalized by the psychotic-like SPQ subscales and the PAGE-R subscales OB, DAP, and HAP were correlated in order to check for excessive collinearity (Field, 2009). Further, in order to test the hypotheses that (1) most PLE are positively associated with other subclinical symptoms whereas (2) magical ideation, paranormal beliefs, and odd beliefs are negatively associated with physical anhedonia, all measures for PLE were correlated with negative-like symptoms, affective symptoms, and other subclinical symptoms. We applied Spearman's correlations, as most variables were ordinal and not normally distributed. We corrected for multiple comparisons using the False Discovery Rate (FDR) algorithm by Benjamini and Hochberg (1995).

#### Regression analyses

Hierarchical multiple linear regression analyses were applied to detect specific associations between different types of PLE (predictors) and outcome variables related to negative-like symptoms (PAS, SPQ no close friends, and constricted affect), affective symptoms (SCL-90-R anxiety, phobic anxiety, obsessive-compulsive, somatization, SPQ social anxiety, and SCL-90-R depression) and other co-occurring symptoms (SCL-90-R interpersonal sensitivity, emotional instability, unspecific symptoms). The MIS was not used as a predictor in the regression models as it includes several different constructs (Vyse, 1997). The control variables age, sex, and educational achievement were entered as predictors in the first block and the psychotic-like subscales of the SPQ followed in the second block. The novel PAGE-R subscales OB, DAP, and HAP (Unterrassner et al., 2017) were added in a third step to test whether they explained variance in addition to the well-established SPQ subscales. All variables were z-standardized in order to make them directly comparable. The predictors were grand mean centered to reduce collinearity. Cases with a standardized residual above absolute 3.29 were removed because values exceeding this threshold are very unlikely to happen by chance in an average sample (Field, 2009). Moreover, the influence of these cases in the resulting regression model may be too strong, especially at smaller sample sizes. Outliers were removed from the models predicting constricted affect (1), phobic anxiety (5), obsessive-compulsive (3), somatization (1), interpersonal sensitivity (2), anger-hostility (7), and additional items (1). Further tests indicated that multicollinearity among predictors was not a concern (*r* < 0.80; tolerance > 0.1; VIF < 10; Myers, 1990; Menard, 1995; Field, 2009) and the data met the assumptions of independent errors (Durbin-Watson value < 3 and >1) and non-zero variances in all models. The scatterplot of standardized predicted values showed that the data met the assumptions of homogeneity of variance and linearity in all cases (Field, 2009). We corrected for multiple comparisons using the FDR algorithm by Benjamini and Hochberg (1995).

## Results

The descriptive statistics of the psychometric instruments are presented in Table 1. We reported some of the correlational analyses already earlier (Unterrassner et al., 2017). These included correlations of the psychotic-like SPQ subscales and the PAGE-R subscales with physical anhedonia, no close friends, constricted affect (Table 2), and social anxiety (Table 3). Moreover, the correlations between psychotic-like SPQ subscales and PAGE-R subscales (Supplementary Table 2).

**Table 1:** Descriptive statistics.

**Variable**	**Median**	**Mean (*SD*)**	**Range**
Age	30	33.11 (11.23)	20–60
Education	3	3.11 (0.97)	0–4
SPQ total score	17	18.86 (10.56)	0–66
PAGE-R total score	15	20.36 (17.63)	0–90
SCL-90-R GSI	0.37	0.46 (0.31)	0–1.52
**PSYCHOTIC-LIKE EXPERIENCES**			
SPQ ideas of reference	3	2.93 (2.24)	0–9
SPQ paranormal beliefs	2	2.44 (2.19)	0–7
SPQ unusual perceptual experiences	2	2.12 (1.97)	0–9
SPQ suspiciousness	2	2.02 (1.71)	0–8
PAGE-R odd beliefs	7	9.52 (8.23)	0–36
PAGE-R dissociative anomalous perceptions	1	2.31 (3.02)	0–22
PAGE-R hallucinatory anomalous perceptions	2	3.88 (4.50)	0–22
**NEGATIVE-LIKE SYMPTOMS**			
Physical Anhedonia Scale	9	10.07 (6.25)	0–30
SPQ no close friends	1	1.36 (1.64)	0–7
SPQ constricted affect	1	1.56 (1.59)	0–7
**AFFECTIVE SYMPTOMS**			
SCL-90-R anxiety	3	3.88 (3.55)	0–16
SCL-90-R phobic anxiety	0	1.09 (1.64)	0–12
SCL-90-R obsessive-compulsive	5	6.21 (5.16)	0–29
SCL-90-R somatization	4	4.85 (4.47)	0–24
SPQ social anxiety	1	1.74 (1.66)	0–7
SCL-90-R depression	6	8.20 (6.69)	0–28
**OTHER SUBCLINICAL SYMPTOMS**			
SCL-90-R interpersonal sensitivity	3	4.12 (4.13)	0–22
SCL-90-R emotional instability	2	2.60 (2.60)	0–13
SCL-90-R unspecific symptoms	4	4.80 (3.32)	0–18

*SPQ, Schizotypal Personality Questionnaire; PAGE-R, Revised Exceptional Experiences Questionnaire; SCL-90-R, Symptom Checklist 90 Revised; GSI, Global Severity Index; N = 206, three cases were missing in the Physical Anhedonia Scale data. Emotional instability was assessed using the SCL-90-R subscale anger-hostility*.

**Table 2:** Spearman's correlation matrix of psychotic-like experiences and negative-like symptoms.

**Psychotic-like experiences**	***r_s_* [CI 95%], *p***		
	**Physical Anhedonia**	**SPQ**	**SPQ**
	**Scale**	**no close friends**	**constricted affect**
SPQ ideas of reference	−0.07 [−0.21, 0.06], 0.292	0.02 [−0.12, 0.15], 0.816	0.07 [−0.07, 0.20], 0.333
SPQ paranormal beliefs	−**0.19 [**−**0.32**, −**0.05], 0.007**	−0.01 [−0.15, 0.13], 0.860	−0.06 [−0.20, 0.08], 0.388
SPQ unusual perceptual experiences	−0.06 [−0.19, 0.08], 0.436	0.08 [−0.06, 0.21], 0.266	0.12 [−0.02, 0.25], 0.090
SPQ suspiciousness	**0.32 [0.19, 0.44], 0.000**	**0.31 [0.18, 0.43], 0.000**	**0.32 [0.19, 0.44], 0.000**
Magical Ideation Scale	−**0.21 [**−**0.33**, −**0.07], 0.003**	0.05 [−0.09, 0.18], 0.518	0.09 [−0.05, 0.22], 0.223
PAGE-R odd beliefs	−0.12 [−0.26, 0.02],0.083	0.05 [−0.08, 0.19], 0.444	0.11 [−0.03, 0.24], 0.135
PAGE-R dissociative anomalous perceptions	−0.07 [−0.21, 0.07], 0.310	*0.12 [*−*0.01, 26], 0.076*	*0.13 [0.00, 0.27], 0.057*
PAGE-R hallucinatory anomalous perceptions	0.00 [−0.14, 0.14], 0.979	0.06 [−0.08, 0.19], 0.416	**0.18 [0.05,0.31], 0.009**

*r_s_, Spearman's rho; CI, Confidence Interval; p, probability; SCL-90-R, Revised Symptom Checklist 90; SPQ, Schizotypal Personality Questionnaire; PAGE-R, Revised Exceptional Experiences Questionnaire. The critical thresholds provided by the FDR procedure (Benjamini and Hochberg, 1995) were 0.077 (α = 0.10, trend), 0.036 (α = 0.05, significant), and 0.006 (α = 0.01, highly significant). Significant correlations are in boldface, statistical trends in italics*.

**Table 3:** Correlation matrix of psychotic-like experiences and affective symptoms.

**Psychotic-like experiences**	***r_s_* [CI 95%], *p***		
	**SCL-90-R**	**SCL-90-R**	**SCL-90-R**
	**anxiety**	**phobic anxiety**	**obsessive-compulsive**
SPQ ideas of reference	**0.39 [0.27, 0.50], 0.000**	**0.33 [0.20, 0.45], 0.000**	**0.37 [0.25, 0.48], 0.000**
SPQ paranormal beliefs	**0.23 [0.10, 0.36], 0.001**	**0.15 [0.02, 0.28], 0.030**	*0.13 [0.00, 0.27], 0.055*
SPQ unusual perceptual experiences	**0.45 [0.34, 0.55], 0.000**	**0.34 [0.21, 0.45], 0.000**	**0.36 [0.23, 0.47], 0.000**
SPQ suspiciousness	**0.23 [0.10, 0.36], 0.001**	**0.26 [0.13, 0.39], 0.000**	**0.28 [0.15, 0.40], 0.000**
Magical Ideation Scale	**0.38 [0.25, 0.49], 0.000**	**0.34 [0.21, 0.46], 0.000**	**0.28 [0.15, 0.41], 0.000**
PAGE-R odd beliefs	**0.35 [0.22, 0.46], 0.000**	**0.29 [0.16, 0.41], 0.000**	**0.29 [0.16, 0.41], 0.000**
PAGE-R dissociative anomalous perceptions	**0.42 [0.31,00.53], 0.000**	**0.31 [0.18, 0.43], 0.000**	**0.30 [0.17, 0.42], 0.000**
PAGE-R hallucinatory anomalous perceptions	**0.33 [0.20, 0.45], 0.000**	**0.29 [0.16, 0.41], 0.000**	**0.31 [0.18, 0.43], 0.000**
	**SCL-90-R**	**SPQ**	**SCL-90-R**
	**somatization**	**social anxiety**	**depression**
SPQ ideas of reference	**0.31 [0.18, 0.43], 0.000**	**0.18 [0.04, 0.31], 0.012**	**0.39 [0.27,0.50], 0.000**
SPQ paranormal beliefs	**0.29 [0.16, 0.41], 0.000**	0.04 [−0.10, 0.18], 0.547	**0.24 [0.11, 0.37], 0.000**
SPQ unusual perceptual experiences	**0.34 [0.21, 0.45], 0.000**	0.05 [−0.09, 0.19], 0.481	**0.35 [0.22, 0.46], 0.000**
SPQ suspiciousness	**0.24 [0.11, 0.37], 0.001**	**0.19 [0.06, 0.32], 0.006**	**0.32 [0.19, 0.43], 0.000**
Magical Ideation Scale	**0.35 [0.22, 0.46], 0.000**	**0.16 [0.02, 0.29], 0.022**	**0.33 [0.20, 0.45], 0.000**
PAGE-R odd beliefs	**0.39 [0.27, 0.50], 0.000**	0.11 [−0.03, 0.24], 0.122	**0.29 [0.15, 0.41], 0.000**
PAGE-R dissociative anomalous perceptions	**0.31 [0.18, 0.43], 0.000**	0.10 [−0.04, 0.23], 0.163	**0.30 [0.17, 0.42], 0.000**
PAGE-R hallucinatory anomalous perceptions	**0.40 [0.28, 0.51], 0.000**	*0.14 [0.00, 0.27], 0.044*	**0.34 [0.21, 0.45], 0.000**

*r_s_, Spearman's rho; CI, confidence interval; p, probability; SCL-90-R, Revised Symptom Checklist 90; SPQ, Schizotypal Personality Questionnaire; PAGE-R, Revised Exceptional Experiences Questionnaire. The critical thresholds provided by the FDR procedure (Benjamini and Hochberg, 1995) were 0.077 (α = 0.10, trend), 0.036 (α = 0.05, significant), and 0.006 (α = 0.01, highly significant). Significant correlations are in boldface, statistical trends in italics*.

### Correlational analysis of psychotic-like experiences (PLE)

As reported earlier (Unterrassner et al., 2017), all PLE were positively associated ranging from weak to strong correlations (see Supplementary Table 2, for the correlation matrix). The weakest correlation was detected between paranormal beliefs and suspiciousness (*r*_*s*_ = 0.19, *p* = 0.006, 95% CI [0.05, 0.32]). The strongest correlation was found between paranormal beliefs and odd beliefs (*r*_*s*_ = 0.69, *p* = 0.000, 95% CI [0.61, 0.75]).

### Correlational analysis of PLE with negative-like symptoms, affective symptoms, and other subclinical symptoms

PLE were tested for significant associations with negative-like symptoms, affective symptoms, and other subclinical symptoms (interpersonal sensitivity, emotional instability, unspecific symptoms). For overview purposes the correlational analyses were grouped into negative-like symptoms (see Table 2, for the correlation matrix), affective symptoms (see Table 3, for the correlation matrix), and other subclinical symptoms (see Table 4, for the correlation matrix). Unless otherwise stated, correlations referred to hereafter were positive.

**Table 4:** Correlation matrix of psychotic-like experiences and other subclinical symptoms.

**Psychotic-like experiences**	***r_s_* [CI 95%], *p***		
	**SCL-90-R**	**SCL-90-R**	**SCL-90-R**
	**interpersonal sensitivity**	**emotional instability**	**unspecific symptoms**
SPQ ideas of reference	**0.43 [0.31, 0.53], 0.000**	**0.29 [0.16, 0.41], 0.000**	0.11 [−0.03, 0.24], 0.115
SPQ paranormal beliefs	**0.19 [0.05, 0.31], 0.008**	0.10 [−0.03, 0.24], 0.142	0.*14 [0.00, 0.27], 0.050*
SPQ unusual perceptual experiences	**0.31 [0.18, 0.43], 0.000**	**0.29 [0.15, 0.41], 0.000**	**0.25 [0.12, 0.38], 0.000**
SPQ suspiciousness	**0.40 [0.28, 0.51], 0.000**	**0.26 [0.13, 0.39], 0.000**	**0.25 [0.12, 0.37], 0.000**
Magical Ideation Scale	**0.38 [0.26, 0.50], 0.000**	**0.27 [0.13, 0.39], 0.000**	**0.20 [0.06, 0.33], 0.005**
PAGE-R odd beliefs	**0.31 [0.18, 0.43], 0.000**	**0.21 [0.07, 0.33], 0.003**	**0.17 [0.04, 0.30], 0.012**
PAGE-R dissociative anomalous perceptions	**0.36 [0.23, 0.47], 0.000**	**0.16 [0.02, 0.29], 0.023**	**0.21 [0.08, 0.34], 0.002**
PAGE-R hallucinatory anomalous perceptions	**0.39 [0.26, 0.50], 0.000**	**0.27 [0.13, 0.39], 0.000**	**0.30 [0.17, 0.42], 0.000**

*r_s_, Spearman's rho; CI, confidence interval; p, probability; SCL-90-R, Revised Symptom Checklist 90; SPQ, m Schizotypal Personality Questionnaire; PAGE-R, Revised Exceptional Experiences Questionnaire. Emotional instability was assessed using the SCL-90-R subscale anger-hostility. The critical thresholds provided by the FDR procedure (Benjamini and Hochberg, 1995) were 0.077 (α = 0.10, trend), 0.036 (α = 0.05, significant), and 0.006 (α = 0.01, highly significant). Significant correlations are in boldface, statistical trends in italics*.

#### Correlational analysis of PLE with negative-like symptoms

Suspiciousness (SPQ) was weakly associated with negative-like symptoms, i.e., the PAS, no close friends (SPQ), and constricted affect (SPQ; see Table 2). Further, hallucinatory anomalous perceptions (PAGE-R) were weakly associated with constricted affect. Moreover, dissociative anomalous perceptions (PAGE-R) trended toward a weak correlation with no close friends and constricted affect. Interestingly, the MIS and paranormal beliefs (SPQ) correlated negatively with the PAS.

#### Correlational analysis of PLE with affective symptoms

All subscales of PLE were weakly to moderately associated with the SCL-90-R subscales anxiety, phobic anxiety, obsessive-compulsive, somatization, and depression (see Table 3). However, paranormal beliefs (SPQ) only trended toward a weak correlation with obsessive-compulsive symptoms. The SPQ subscales ideas of reference and suspiciousness, and the MIS were weakly correlated with social anxiety (SPQ). Hallucinatory anomalous perceptions (PAGE-R) trended toward correlating positively with social anxiety (SPQ). We did not detect any negative associations of PLE and affective symptoms.

#### Correlational analysis of PLE with other subclinical symptoms

PLE correlated weakly to moderately with the SCL-90-R subscales interpersonal sensitivity, emotional instability (except SPQ paranormal beliefs), and unspecific symptoms (except SPQ ideas of reference; see Table 4). SPQ paranormal beliefs trended toward correlating weakly with unspecific symptoms. We did not detect any negative correlations of PLE and other subclinical symptoms.

In summary, the correlational analyses indicated that PLE in healthy individuals were weakly to moderately associated with various negative-like symptoms, affective symptoms, and other subclinical symptoms. Notably, we found that magical ideation and paranormal beliefs were negatively correlated with physical anhedonia.

### Regression analyses predicting subclinical symptoms from PLE

Hierarchical multiple linear regression models were used to uncover unique associations between different types of PLE and subclinical symptoms while adjusting for age, sex, and education. Unless otherwise stated, the standardized regression weights reported hereafter were positive.

#### Prediction of negative-like symptoms from PLE

The hierarchical multiple linear regression models predicting negative-like symptoms are displayed in Table 5. Suspiciousness (SPQ) was a unique positive predictor of physical anhedonia, no close friends, and constricted affect. Notably, ideas of reference negatively predicted physical anhedonia, and paranormal beliefs negatively predicted constricted affect.

**Table 5:** Regression analyses predicting negative-like symptoms from psychotic-like experiences.

**Predictor**	**Physical Anhedonia Scale**		**SPQ no close friends**		**SPQ constricted affect**	
	**ΔR^2^, *p***	**β [95% CI], *p***	**ΔR^2^, *p***	**β [95% CI], *p***	**ΔR^2^, *p***	**β [95% CI], *p***
Step 1	**0.08, 0.001**		0.01, 0.719		0.01, 0.491	
Control variables^a^						
Step 2	**0.15, 0.000**		**0.14, 0.000**		**0.15, 0.000**	
SPQ ideas of reference		−**0.22 [**−**0.38**, −**0.06], 0.009**		−0.11 [−0.28, 0.06], 0.210		−0.02 [−0.20, 0.15], 0.782
SPQ paranormal beliefs		−0.08 [−0.24, 0.08], 0.310		−0.08 [−0.25, 0.09], 0.344		−**0.23 [**−**0.39**, −**0.06], 0.008**
SPQ unusual perceptual experiences		−0.09 [−0.26, 0.07], 0.251		0.10 [−0.07, 0.27], 0.251		0.14 [−0.03, 0.31], 0.096
SPQ suspiciousness		**0.43 [0.28, 0.58], 0.000**		**0.39 [0.24, 0.55], 0.000**		**0.34 [0.19, 0.50], 0.000**
Step 3	0.01, 0.653		0.01, 0.603		0.01, 0.399	
PAGE-R odd beliefs		−0.13 [−0.36, 0.10], 0.255		0.02 [−0.22, 0.26], 0.646		0.09 [−0.15, 0.33], 0.470
PAGE-R dissociative anomalous perceptions		0.00 [−0.18, 0.18], 0.978		0.12 [−0.07, 0.31], 0.221		0.05 [−0.14, 0.24], 0.572
PAGE-R hallucinatory anomalous perceptions		0.00 [−0.18, 0.20], 0.930		−0.04 [−0.24, 0.16], 0.729		0.06 [−0.14, 0.26], 0.568
Total *R^2^*	0.24		0.16		0.18	
*F*-test	*F*_(10, 192)_ = 6.173, *p* = 0.000		*F*_(10, 195)_ = 3.593, *p* = 0.000		*F*_(10, 194)_ = 4.104, *p* = 0.000	
*n*	203		206		205	

a *Control variables include age, sex, and education. Physical anhedonia lacked three cases. One outlier was removed from the model predicting constricted affect. SPQ, Schizotypal Personality Questionnaire; PAGE-R, Revised Exceptional Experiences Questionnaire. The critical thresholds provided by the FDR procedure (Benjamini and Hochberg, 1995) were 0.030 (α = 0.10, trend), 0.013 (α = 0.05, significant), and 0.001 (α = 0.01, highly significant). Significant values are in boldface*.

#### Prediction of affective symptoms from PLE

The regression models predicting affective symptoms are depicted in Table 6. Interestingly, a similar pattern of specific associations was detected across the models predicting symptoms related to anxiety from PLE: Ideas of reference, unusual perceptual experiences, and dissociative anomalous perceptions were often significant predictors of, or trended toward positively predicting anxiety, phobic anxiety, obsessive-compulsive symptoms, and somatization. This pattern was even more evident and also included the regression model predicting social anxiety when correction for multiple comparisons was not taken into account. The SCL-90-R depression subscale was specifically predicted by ideas of reference (SPQ), whereas odd beliefs (PAGE-R) negatively predicted depressive symptoms.

**Table 6:** Regression analyses predicting affective symptoms from psychotic-like experiences.

**Predictor**	**SCL90-R anxiety**		**SCL-90-R phobic anxiety**		**SCL-90-R obsessive-compulsive**	
	**Δ*R*^2^, *p***	**β [95% CI], *p***	**Δ*R*^2^, *p***	**β [95% CI], *p***	**Δ*R*^2^, *p***	**β [95% CI], *p***
Step 1	**0.10, 0.000**		−0.02, 0.181		**0.15, 0.000**	
Control variables^a^						
Step 2	**0.22, 0.000**		−**0.21, 0.000**		**0.22, 0.000**	
SPQ ideas of reference		0.16 [0.01, 0.31], 0.040		**0.28 [0.11, 0.44], 0.001**		**0.21 [0.06, 0.36], 0.006**
SPQ paranormal beliefs		0.09 [−0.06, 0.24], 0.252		0.01 [−0.16, 0.17], 0.953		0.01 [−0.14, 0.15], 0.922
SPQ unusual perceptual experiences		**0.34 [0.19, 0.49], 0.000**		**0.26 [0.10, 0.43], 0.002**		**0.31 [0.16, 0.46], 0.000**
SPQ suspiciousness		−0.04 [−0.18, 0.10], 0.579		−0.01 [−0.16, 0.14], 0.927		0.03 [−0.10, 0.17], 0.652
Step 3	*0.04, 0.013*		**0.11, 0.000**		0.02, 0.116	
PAGE-R odd beliefs		−0.11 [−0.33, 0.10], 0.292		0.04 [−0.18, 0.25], 0.736		−0.14 [−0.35, 0.07], 0.176
PAGE-R dissociative anomalous perceptions		**0.28 [0.11, 0.44], 0.001**		**0.45 [.28, 0.63], 0.000**		*0.19 [0.03, 0.35], 0.025*
PAGE-R hallucinatory anomalous perceptions		−0.06 [−0.23, 0.12], 0.531		−0.15 [−0.34, 0.04], 0.119		−0.02 [−0.20, 0.16], 0.821
Total *R*^2^	0.36		0.34		0.39	
*F*-test	*F*_(10, 195)_ = 10.799, *p* = 0.000		*F*_(10, 190)_ = 9.900, *p* = 0.000		*F*_(10, 192)_ = 12.168, *p* = 0.000	
*n*	206		201		203	
**Predictor**	**SCL-90-R somatization**		**SCL-90-R social anxiety**		**SCL-90-R depression**	
	Δ***R***^2^, ***p***	β **[95% CI]**, ***p***	Δ***R***^2^, ***p***	β **[95% CI]**, ***p***	Δ***R***^2^, ***p***	β **[95% CI]**, ***p***
Step 1	**0.11, 0.000**		**0.13, 0.000**		**0.13, 0.000**	
Control variables^a^						
Step 2	**0.13, 0.000**		0.04, 0.070		**0.17, 0.000**	
SPQ ideas of reference		0.12 [−0.04, 0.28], 0.151		0.16 [−0.01, 0.33], 0.070		**0.20 [0.05, 0.36], 0.012**
SPQ paranormal beliefs		0.15 [−0.01, 0.30], 0.072		0.02 [−0.14, 0.19], 0.772		0.13 [−0.03, 0.28], 0.107
SPQ unusual perceptual experiences		*0.18 [0.02, 0.34], 0.026*		−0.09 [−0.25, 0.08], 0.315		0.10 [−0.06, 0.25], 0.222
SPQ suspiciousness		0.03 [−0.12, 0.18], 0.692		0.10 [−0.06, 0.25], 0.207		0.13 [−0.01, 0.27], 0.062
Step 3	0.02, 0.146		0.02, 0.189		*0.03, 0.027*	
PAGE-R odd beliefs		−0.10 [−0.32, 0.13], 0.402		−0.13 [−0.37, 0.11], 0.295		−**0.31 [**−**0.53**, −**0.09], 0.005**
PAGE-R dissociative anomalous perceptions		0.18 [0.00, 0.36], 0.049		0.20 [0.01, 0.39], 0.036		0.14 [−0.03, 0.31], 0.101
PAGE-R hallucinatory anomalous perceptions		0.04 [−0.15, 0.23], 0.669		−0.07 [−0.27, 0.12], 0.469		0.08 [−0.09, 0.26], 0.357
Total *R*^2^	0.27		0.19		0.33	
*F*-test	*F*_(10, 194)_ = 7.019, *p* = 0.000		*F*_(10, 195)_ = 4.465, *p* = 0.000		*F*_(10, 195)_ = 9.683, *p* = 0.000	
*n*	205		206		206	

a *Control variables include age, sex, and education. Five outliers were removed from the model predicting phobic anxiety, three from the model predicting obsessive-compulsive symptoms, and one from the model predicting somatization. SCL-90-R, Revised Symptom Checklist 90; SPQ, Schizotypal Personality Questionnaire; PAGE-R, Revised Exceptional Experiences Questionnaire. The critical thresholds provided by the FDR procedure (Benjamini and Hochberg, 1995) were 0.030 (α = 0.10, trend), 0.013 (α = 0.05, significant), and 0.001 (α = 0.01, highly significant). Significant values are in boldface, statistical trends in italics*.

#### Prediction of other subclinical symptoms from PLE

The regression models predicting additional psychopathological symptoms are presented in Table 7. The SCL-90-R subscale interpersonal sensitivity was uniquely predicted by ideas of reference, suspiciousness (SPQ), and dissociative anomalous perceptions (PAGE-R). SCL-90-R emotional instability was predicted by ideas of reference and unusual perceptual experiences (SPQ). Unspecific symptoms (SCL-90-R additional items) were specifically predicted by dissociative anomalous perceptions (PAGE-R). Interestingly, odd beliefs (PAGE-R) were a negative predictor of emotional instability and unspecific symptoms. Moreover, emotional instability was also negatively predicted by paranormal beliefs (SPQ), unless corrected for multiple comparisons.

**Table 7:** Regression analyses predicting other subclinical symptoms from psychotic-like experiences.

**Predictor**	**SCL-90-R interpersonal sensitivity**		**SCL-90-R emotional instability**		**SCL-90-R unspecific symptoms**	
	**Δ*R*^2^, *p***	**β [95% CI], *p***	**Δ*R*^2^, *p***	**β [95% CI], *p***	**Δ*R*^2^, *p***	**β [95% CI], *p***
Step 1	**0.08, 0.001**		**0.09, 0.000**		*0.04, 0.035*	
Control variables^a^						
Step 2	**0.26, 0.000**		**0.15, 0.000**		**0.06, 0.012**	
SPQ ideas of reference		**0.31 [0.16, 0.46], 0.000**		**0.25 [0.08, 0.41], 0.004**		−0.06 [−0.23, 0.12], 0.527
SPQ paranormal beliefs		0.02 [−0.13, 0.17], 0.788		−0.18 [−0.34, −0.02], 0.032		0.06 [−0.12, 0.23], 0.517
SPQ Unusual perceptual experiences		0.10 [−0.05, 0.25], 0.183		**0.22 [0.06, 0.38], 0.009**		0.16 [−0.01, 0.33], 0.072
SPQ suspiciousness		**0.21 [0.08, 0.35], 0.002**		0.10 [−0.05, 0.24], 0.208		0.15[−0.01, 0.31], 0.072
Step 3	**0.07, 0.000**		0.04, 0.026		**0.07, 0.001**	
PAGE-R odd beliefs		−0.03 [−0.24, 0.17], 0.740		−**0.29 [**−**0.52**, −**0.07], 0.012**		−**0.33 [**−**0.57**, −**0.09], 0.007**
PAGE-R dissociative anomalous perceptions		**0.31 [0.15, 0.47], 0.000**		−0.05 [−0.23, 0.13], 0.611		**0.25 [0.06, 0.44], 0.010**
PAGE-R hallucinatory anomalous perceptions		0.07 [−0.10, 0.24], 0.405		*0.23 [0.04, 0.43], 0.017*		0.18 [−0.02, 0.37], 0.083
Total *R*^2^	0.41		0.28		0.17	
*F*-test	*F*_(10, 193)_ = 13.284, *p* = 0.000		*F*_(10, 188)_ = 7.231, *p* = 0.000		*F*_(10, 194)_ = 3.991, *p* = 0.000	
*n*	204		199		205	

a *Control variables include age, sex, and education. Emotional instability was assessed using the anger-hostility subscale of the SCL-90-R. Two outliers were removed from the model predicting interpersonal sensitivity, seven from the model predicting emotional instability (anger-hostility), and one from the model predicting additional items. SCL-90-R, Revised Symptom Checklist 90; SPQ, Schizotypal Personality Questionnaire; PAGE-R, Revised Exceptional Experiences Questionnaire. The critical thresholds provided by the FDR procedure (Benjamini and Hochberg, 1995) were.030 (α = 0.10, trend), 0.013 (α = 0.05, significant), and 0.001 (α = 0.01, highly significant). Significant values are in boldface, statistical trends in italics*.

In summary, we found similar patterns of positive associations of PLE among negative-like symptoms and anxiety symptoms in healthy individuals. Notably, a number of delusional-like experiences negatively predicted negative-like symptoms, depressive symptoms, emotional instability and unspecific symptoms. However, we could not detect any negative associations of PLE with anxiety symptoms.

## Discussion

To the best of our knowledge this is the first study to investigate specific associations between subtypes of PLE and negative-like symptoms, affective symptoms, and other subclinical symptoms in a sample of healthy individuals. Consistent with our primary hypotheses and earlier findings, we found that (1) most PLE correlated positively with affective symptoms and other subclinical symptoms, whereas they only partially correlated with negative-like symptoms. As hypothesized, (2) higher scores on magical ideation and paranormal beliefs were associated with lower scores on physical anhedonia. Interestingly, in the regression models predicting subclinical symptoms from PLE, we found (3) similar associations among the variables in each model to positively predict negative-like symptoms and anxiety symptoms. Similar to the findings of Yung et al. (2006), (4) a number of delusional-like symptoms negatively predicted negative-like symptoms, depressive symptoms, emotional instability, and unspecific symptoms. Our results suggest that subtypes of PLE might specifically be associated with negative-like symptoms, affective symptoms, and other subclinical symptoms. Moreover, our findings might indicate that not all PLE are necessarily associated with psychological burden. Further, some delusional-like PLE might even be beneficial for subjective well-being, despite conferring an inaccurate representation of the world. The results of this study might be a starting point toward investigating a more fine-grained interplay of PLE and other subclinical symptoms in longitudinal studies.

### Delusional aspects of PLE are inversely correlated with physical anhedonia

In an earlier study (Unterrassner et al., 2017), we showed that even in healthy individuals, suspiciousness (SPQ) and schizotypal signs (Rössler et al., 2007) were positively correlated with negative-like symptoms (physical anhedonia, no close friends, constricted affect) and social anxiety. Here, we expanded these findings by showing that PLE as measured by the SPQ (Raine et al., 1994) and the PAGE-R were also positively correlated with other subclinical psychopathological symptoms as depicted by the SCL-90-R (see Tables 4, 5). These results were in line with our first initial hypothesis that most PLE are positively correlated with co-morbid symptoms not only in clinical and general population samples (see Linscott and van Os, 2013) but also at the healthy end of the psychosis continuum. Importantly, our results are in line with the notion that psychosis constitutes a phenotype that is continuously distributed in the general population (Johns and van Os, 2001).

Confirming our second hypothesis, we found negative correlations between the PAS and the MIS. This result consolidates our earlier observation that physical anhedonia negatively correlated with paranormal beliefs (SPQ) and odd beliefs (PAGE-R, trend; Unterrassner et al., 2017). These findings are in line with those of Chapman et al. (1982) who repeatedly demonstrated negative correlations between the PAS and the MIS as well as the Perceptual Aberration Scale (Chapman et al., 1978). Further analyses by the latter authors indicated that the negative correlation was likely to reflect a true mutual incompatibility of physical anhedonia and PLE instead of an artificial result of e.g., a sampling effect (Chapman et al., 1982). However, in a clinical sample and schizophrenic patients, the negative relationship between these scales was not detected (Chapman et al., 1978). The authors presumed that high-scoring individuals on both traits tend to become clinical patients or hospitalized more often than low scorers. Consequently, co-occurring high-scores on physical anhedonia and PLE in a clinical sample might cancel out their otherwise negative correlation. Similarly, Yung et al. (2006) found that magical thinking was not correlated with depression in help-seeking young people with a non-psychotic disorder, unless accompanied by distress. As we examined healthy individuals, we expected magical ideation, paranormal beliefs, and odd beliefs to be also negatively correlated with the SCL-90-R subscale depression. Instead, they proved to be *positively* correlated. In the light of their negative correlation with physical anhedonia, this finding was surprising. However, it might be explained by the fact that the subscale used by Yung et al. (2006) to assess depression mainly inquired anhedonic symptoms (anhedonic depression scale of the Mood and Anxiety Questionnaire; Watson and Clark, 1991). This contrasts with the depression subscale of the SCL-90-R that we used, which only contains one item referring to anhedonia (reduced interest or pleasure in sexuality) and mainly covers other symptoms, such as, loss of motivation and energy or dysphoric mood and feelings (Schmitz et al., 2000). Therefore, our results might indicate that magical ideation, paranormal beliefs, and odd beliefs are only negatively correlated with physical anhedonia but are positively correlated with other depressive symptoms relating to negative feelings and sadness.

The correlational analyses suggested that also in healthy individuals PLE might be seen as indicators for reduced psychological functioning. However, we have evidence supporting earlier findings (Chapman et al., 1982; Yung et al., 2006), indicating that not all forms of PLE are equally and necessarily associated with other subclinical difficulties. Particularly PLE relating to magical ideation, paranormal beliefs, or odd beliefs might be accompanied by lower physical anhedonia or an increased ability to enjoy sensory stimuli, respectively. Possible explanations for negative relationships between delusional PLE and other symptoms are discussed below (see Section Delusional-Like Symptoms Negatively Predict Other Subclinical Symptoms).

### Similar aspects of PLE are associated with negative-like symptoms and anxiety symptoms

In this study, we found consistent patterns of *positive* associations across the models predicting negative-like symptoms and anxiety symptoms, respectively. Importantly, the patterns did not overlap between the two symptom clusters. This means suspiciousness was only implicated in negative-like symptoms, while ideas of reference, unusual perceptual experiences, and dissociative anomalous perceptions were only associated with anxiety symptoms. Similar to anxiety symptoms, depressive symptoms were also specifically predicted by ideas of reference.

Our results indicated that suspiciousness in healthy individuals is specifically linked to a reduced experience (physical anhedonia, social anhedonia) and expression (constricted affect) of emotions. Suspiciousness is the less extreme version of paranoia and involves the exaggerated tendency to believe that other people intend harm (Lenzenweger et al., 1997). Importantly, emotions may serve as a source of information as well as a heuristic to make decisions, judgments, and attributions (Wilson and Gilbert, 2005). Understanding their emotions might help individuals to identify and respond in an appropriate manner to observations that elicited their emotions. In this context, individuals scoring high on these negative-like symptoms might be challenged to appropriately evaluate social cues. This in turn might entail insecurities regarding social interactions and negative expectations. Fittingly, Boden and Berenbaum (2012) found evidence that individuals with diminished emotional clarity and diminished awareness of the source of their emotions are indeed at increased risk for suspiciousness.

The results indicated that ideas of reference might specifically be implicated in affective difficulties, i.e., anxiety symptoms and depressive symptoms. This result could be explained by the observation that the SCL-90-R depression subscale mainly covers experiences referring to loss of motivation, dysphoric mood, and sadness (Schmitz et al., 2000), which might also be related to anxiety problems: Several studies have demonstrated that self-referential thinking is fostered by negative emotions (Salovey, 1992; Rochat et al., 2012) and it might intensify negative affect and maintain depression (Pyszczynski and Greenberg, 1987).

There might be a causal explanation for the specific associations between anxiety symptoms and unusual perceptual experiences and dissociative anomalous perceptions, respectively. In a recent review on normal and dysregulated interoception in mental disorders, Schulz and Vögele (2015) proposed a model, in which anxiety and acute stress are implicated in an altered perception of bodily sensations and the generation of subjectively perceived physical symptoms, i.e., somatization symptoms. Similarly, stress sensitivity has recently been discussed as a factor that might be involved in the emergence of PLE in the general population (Gibson et al., 2014). However, the specific associations between anomalous perceptions and anxiety symptoms might also be based on a conceptual overlap between these constructs. For example, the anxiety subscale and the somatization subscale capture anomalous perceptions in the sense of bodily symptoms and somatic manifestations of anxiety that lack any apparent cause. Moreover, the obsessive-compulsive subscale covers thoughts, impulses, and actions that are perceived as being unwanted, unchangeable, and alien to the self (cf. dissociative anomalous perceptions). Indeed, it has been noted before that the difference between people with anxiety-related difficulties and psychotic symptoms are mainly (but not only) related to their cultural acceptability: For example, immediately interpreting a benign lump on the skin as cancer may qualify as a hypochondriac symptom. In contrast, taking this observation as evidence for a transmitter implanted by the secret police may be classified as a psychotic symptom (Morrison et al., 2004). Hence, both, anxiety symptoms and psychotic symptoms might represent instances where increased salience is assigned to bodily perceptions (Kapur, 2003) whereas they differ in the way they are interpreted.

The presently reported consistency of specific associations between PLE and negative-like symptoms as well as anxiety symptoms across different regression models and psychometric measures is intriguing. Although we cannot draw any causal conclusion based on the present data, our findings might point toward possible causal pathways between PLE and associated psychological difficulties. Importantly, they indicate that individuals might benefit from therapeutic measures addressing specific symptoms. For example, training emotional clarity and source awareness might help to reduce paranoid symptoms and prevent their exacerbation (Boden and Berenbaum, 2012).

### Specific positive associations of PLE with other subclinical symptoms

In addition to negative-like symptoms and affective symptoms we investigated specific associations between PLE and other subclinical symptoms, i.e., the SCL-90-R subscales interpersonal sensitivity, emotional instability, and unspecific symptoms (additional symptoms). Interpersonal sensitivity refers to an excessive awareness of the behavior and feelings of other people (Boyce and Parker, 1989). The association of interpersonal sensitivity with suspiciousness is likely to be related to the negative expectations and insecurities regarding social interactions that characterize high-scoring individuals on interpersonal sensitivity (Schmitz et al., 2000). Its association with ideas of reference might be explained by the fact that the latter are an inherent characteristic of many experiences referring to interpersonal sensitivity as they mainly base on comparisons of oneself with others. The associations of interpersonal sensitivity with ideas of reference and dissociative anomalous perceptions are reminiscent of the patterns seen in the models predicting anxiety symptoms. Interpersonal sensitivity has been discussed as an underlying personality trait in anxiety disorders, which could explain their similar associations with PLE (Harb et al., 2002; Wilhelm et al., 2004). The SCL-90-R anger-hostility subscale measures irritability and emotional instability through strong aggressiveness. We found emotional instability (anger-hostility) to be positively predicted by ideas of reference and unusual perceptual experiences. Hence, the tendency to refer observations to oneself and a distorted perception of the environment (as depicted by the numerous illusions in the anomalous perceptual experiences subscale) might be specifically implicated in higher emotional irritability or lower emotional stability, respectively. The additional items subscale of the SCL-90-R does not reflect a specific construct but encompasses a variety of rather unspecific difficulties such as, poor appetite or disturbed sleep. Therefore, their positive association with dissociative anomalous perceptions could not readily be interpreted.

### Delusional-like symptoms negatively predict other subclinical symptoms

We found that the PAGE-R subscale odd beliefs as well as the SPQ subscales paranormal beliefs and ideas of reference negatively predicted negative-like symptoms (physical anhedonia, constricted affect), depressive symptoms, emotional instability, and unspecific symptoms (see Tables 6, 7, for the regression models). This means that when all other variables in the models were held constant, higher scores on these delusional-like PLE indicated lower scores on some of the latter problems. Similarly, Yung et al. (2006) found no significant correlation between magical thinking and anhedonic depression unless accompanied by distress. Moreover, magical thinking negatively predicted anhedonic depression in a multivariate regression model (when bizarre experiences and persecutory ideas were included as predictors). These findings were interpreted as indications that paranormal beliefs must not necessarily be maladaptive. Our results further substantiate the findings by Yung et al. (2006) that delusional-like symptoms negatively predict symptoms related to anhedonic depression. Further, they extend their findings by showing that (1) not only paranormal beliefs but also odd beliefs and ideas of reference may show inverse associations with other subclinical symptoms. Moreover, (2) delusional-like symptoms may not only negatively predict symptoms related to anhedonia but also depressive symptoms incorporating e.g., feelings of sadness. As follows, we offer some interpretations of the observed negative associations.

#### Odd beliefs might alleviate symptoms by conferring meaning to one's life

The negative association between odd beliefs and depressive symptoms, emotional instability, and unspecific symptoms might be causally explained. In an earlier study on the same sample, we showed that odd beliefs were particularly associated with positive valence ratings, suggesting a foremost enriching and perhaps comforting quality of these experiences (Unterrassner et al., 2017). Moreover, odd beliefs were specifically associated with waking states, mental techniques, occult practices, and extreme situations, which might indicate that they especially occur in situations where revelations, answers or help are actively sought. It has been suggested earlier that similar experiences might be a means to reduce distress in perceptually ambiguous or stressful situations (Malinowski, 1954; Beitman, 2009), re-establish (perceived) control under lack of control, and create confidence and agency (Whitson and Galinsky, 2008). Hence, it is likely that the tendency to perceive meaning in random events (odd beliefs) facilitates a more positive view about one's position in the world and observations, and thereby attenuates depressive symptoms. As odd beliefs are also positively correlated with depressive symptoms and other psychological difficulties, it might be speculated that they form in response to an increased load of symptoms.

#### Paranormal beliefs might specifically be associated with sociability

The negative associations between paranormal beliefs and constricted affect fits well with the finding that paranormal beliefs are positively associated with the personality traits extraversion and emotional stability (Perdue, 2013). The latter author proposed that extroverted individuals might be more likely to endorse unconventional beliefs as they engage more in social activities and are more open to new experiences. Hence, it might be that the tendency to hold paranormal beliefs is inherently associated with increased sociability and thus, with less difficulties in display of emotions (constricted affect).

#### Positive ideas of reference might come along with the capability to enjoy sensory stimuli

Our results suggested that at a given level of co-occurring PLE, ideas of reference might not only be associated with higher anxiety scores but also with lower scores on physical anhedonia. Whereas, suspiciousness and ideas of reference have exaggerated self-referral in common, ideas of reference may not only be unpleasant (Lenzenweger et al., 1997; Cicero and Kerns, 2011). Notably, the experiences in the ideas of reference subscale that we used are mostly neutrally connoted (see Raine, 1991). As ideas of reference might confer subjective significance of experiences, they might facilitate a more pleasurable experience of the world. Therefore, ideas of reference might be incompatible with the inability to enjoy sensory stimuli (physical anhedonia) to some degree, which might explain its negative association with physical anhedonia in the regression model. However, more differentiated psychometric measures for positive and negative ideas of reference are needed to clarify the exact associations between physical anhedonia and negative, positive, and neutral ideas of reference.

### Toward a differentiated categorization and assessment of PLE

Our regression analyses revealed specific associations between subtypes of PLE and other subclinical symptoms in healthy individuals. Referring to the categorization models proposed by van Os and Reininghaus (2016) and Yung et al. (2009; see Section Introduction) our results suggest that suspiciousness might specifically reflect vulnerability toward psychosis, as this subscale was uniquely associated with one aspect of psychosis, negative symptoms (category 1). In contrast, ideas of reference, unusual perceptual experiences and dissociative anomalous perceptions might be less specific for psychosis and also be implicated in the development of affective disorders (category 2), because they predicted affective symptoms and other subclinical symptoms, but not negative-like symptoms. Lastly, paranormal beliefs and odd beliefs might not necessarily be associated with clinical disorder at all (category 3), as they only negatively predicted subclinical symptoms in the regression models. Whereas, PLE of categories 1 and 2 might specifically be implicated in reduced functioning in healthy individuals, experiences of category 3 might be neutral or even beneficial for subjective well being. Given their positive correlations with different subclinical symptoms, it might be speculated that positively-valenced delusional-like experiences in healthy individuals form in response to an increased load of symptoms and/or distressing life experiences. This means that delusional-like symptoms facilitating the integration of adverse experiences into a positive and stabilizing framework might partly explain why some people maintain functioning despite the presence of PLE and affective symptoms. Although more data is needed to determine which PLE are maladaptive or not (Yung et al., 2009; van Os and Reininghaus, 2016), our findings underline the importance of discerning between different subtypes of PLE. Moreover, our results indicate that the incorporation of measures for positively-valenced delusional-like PLE might prove to be important in the study of subclinical psychosis as studies have often exclusively focused on negatively-valenced beliefs (Wiseman and Watt, 2004; Perdue, 2013).

## Limitations

Several limitations to our study need to be considered. For example, young, male, and well-educated individuals characterized the present sample. In order to improve the generalizability of the findings, future research samples should be representative of the general population. Further, diagnoses and family history of psychotic illness were not verified through diagnostic interviews but by asking the participants. Moreover, the “caseness” criterion rather represents an unspecific pathology and is not a state-of-the-art methodology in capturing mental health states (Mueller et al., 2009). Hence, controlling for psychopathology could be improved by using diagnostic interview tools. As this study was cross-sectional, drawing conclusions about the causal relationships between PLE and other subclinical symptoms was not possible. Therefore, a longitudinal study design should be implemented in future research to clarify their causal relationships. The SPQ, the MIS, and the PAS only included dichotomous items, which may have limited their informative value. Moreover, social anhedonia and manic symptoms were not assessed in the present study although they also reflect aspects of negative-like symptoms or affective dysregulation, respectively. There are several self-report measures that offer themselves for a more comprehensive assessment of anhedonic symptoms and depressive symptoms, such as, the Revised Social Anhedonia Scale Eckblad et al. Unpublished manuscript, the Anticipatory and Consummatory Interpersonal Pleasure Scale (ACIPS; Gooding and Pflum, 2014), the Center for Epidemiological Studies-Depression Scale (CES-D; Radloff, 1977) or the Beck Depression Inventory-II (BDI-II; Beck et al., 1996). Future research might also consider measures that combine the assessment of PLE, negative-like symptoms and depressive symptoms, such as, the Community Assessment of Psychic Experiences questionnaire (CAPE; Stefanis et al., 2002). Lastly, including interview-based assessments might complement the coverage of subclinical symptoms with self-report measures.

## Conclusion

We found consistent patterns of positive multivariate associations of PLE with negative-like symptoms and anxiety symptoms. Notably, the patterns were consistent across several psychometric instruments and separated well between these two symptom clusters. Although these observations support the validity of our findings, it needs to be determined whether they generalize to other populations and psychometric measures as well. The identification of specific relations between subtypes of PLE and other subclinical symptoms might contribute to cognitive models preoccupied with the formation of psychotic and other mental disorders and might help to find new strategies in psychosis prevention. For example, addressing specific symptoms in psychotherapy might more efficiently lower the overall symptom load and counteract the exacerbation of psychopathological symptoms. However, longitudinal studies are needed in order to clarify the causal role of different PLE and other subclinical symptoms in the exacerbation of symptoms or the maintenance of mental health, respectively. Lastly, it needs to be determined whether the positive and negative associations between PLE and psychopathological symptoms are confined to healthy individuals or if they can be found in general population samples or clinical samples as well.

## Author contributions

LU contributed to the acquisition of data, analyzed and interpreted the data, and drafted the manuscript. TW contributed to the acquisition of data and critically revised the manuscript. DW contributed to the interpretation of the data and revised the manuscript critically. HH and WR contributed to the conception and design of the study and revised the manuscript critically. All authors have given final approval for the version to be published and agreed to be accountable for all aspects of the work.

### Conflict of interest statement

The authors declare that the research was conducted in the absence of any commercial or financial relationships that could be construed as a potential conflict of interest. The reviewer JK and handling Editor declared their shared affiliation.
