# Supplementary material for: The Intricate Relationship between Psychotic-Like Experiences and Associated Subclinical Symptoms in Healthy Individuals
Source: Front Psychol. 2017 Sep 7;8:1537. doi: 10.3389/fpsyg.2017.01537 (PMC5594214; doi:10.3389/fpsyg.2017.01537)
Supplement: Supplementary file 1 [file Table1.docx]

Supplementary Material

The Intricate Relationship between Psychotic-Like Experiences and Associated Subclinical Symptoms in Healthy Individuals

Lui Unterrassner^1^*, Thomas Wyss^1^, Diana Wotruba^1^, Vladeta Ajdacic-Gross^2^, Helene Haker^1,3^, and Wulf Rössler^1,2,4^

*** Correspondence:** Corresponding Author: unterrassner@collegium.ethz.ch

**Supplementary Table 1**

**Number of Missing Data Points in the PAGE-R and SCL-90-R Data per Item.** In total, the PAGE-R data was lacking 5 data points (0.07%) and the SCL-90-R data 69 data points (0.32%).

| Item No. | Missing | Missing % |
| --- | --- | --- |
|  |  |  |
| PAGE-R |  |  |
| 8 | 3 | 1.3 |
| 13 | 1 | 0.4 |
| 23 | 1 | 0.4 |
|  |  |  |
| SCL-90-R |  |  |
| 5 | 1 | 0.4 |
| 10 | 1 | 0.4 |
| 19 | 1 | 0.4 |
| 24 | 1 | 0.4 |
| 25 | 3 | 1.3 |
| 28 | 1 | 0.4 |
| 31 | 1 | 0.4 |
| 33 | 1 | 0.4 |
| 36 | 3 | 1.3 |
| 38 | 1 | 0.4 |
| 39 | 1 | 0.4 |
| 40 | 2 | 0.8 |
| 42 | 1 | 0.4 |
| 43 | 1 | 0.4 |
| 49 | 1 | 0.4 |
| 50 | 1 | 0.4 |
| 52 | 3 | 1.3 |
| 54 | 1 | 0.4 |
| 55 | 1 | 0.4 |
| 56 | 1 | 0.4 |
| 59 | 1 | 0.4 |
| 60 | 2 | 0.8 |
| 61 | 1 | 0.4 |
| 62 | 2 | 0.8 |
| 63 | 2 | 0.8 |
| 64 | 4 | 1.7 |
| 65 | 1 | 0.4 |
| 66 | 1 | 0.4 |
| 67 | 1 | 0.4 |
| 68 | 1 | 0.4 |
| 69 | 1 | 0.4 |
| 70 | 1 | 0.4 |
| 71 | 1 | 0.4 |
| 72 | 1 | 0.4 |
| 73 | 1 | 0.4 |
| 74 | 2 | 0.8 |
| 75 | 1 | 0.4 |
| 76 | 1 | 0.4 |
| 77 | 1 | 0.4 |
| 78 | 1 | 0.4 |
| 79 | 1 | 0.4 |
| 80 | 1 | 0.4 |
| 81 | 1 | 0.4 |
| 82 | 1 | 0.4 |
| 83 | 1 | 0.4 |
| 84 | 1 | 0.4 |
| 85 | 2 | 0.8 |
| 86 | 2 | 0.8 |
| 87 | 2 | 0.8 |
| 88 | 1 | 0.4 |
| 89 | 1 | 0.4 |
| 90 | 1 | 0.4 |
