# Supplementary material for: The Intricate Relationship between Psychotic-Like Experiences and Associated Subclinical Symptoms in Healthy Individuals
Source: Front Psychol. 2017 Sep 7;8:1537. doi: 10.3389/fpsyg.2017.01537 (PMC5594214; doi:10.3389/fpsyg.2017.01537)
Supplement: Supplementary file 2 [file Table2.docx]

Supplementary Material

The Intricate Relationship between Psychotic-Like Experiences and Associated Subclinical Symptoms in Healthy Individuals

Lui Unterrassner^1^*, Thomas Wyss^1^, Diana Wotruba^1^, Vladeta Ajdacic-Gross^2^, Helene Haker^1,3^, and Wulf Rössler^1,2,4^

*** Correspondence:** Corresponding Author: unterrassner@collegium.ethz.ch

**Supplementary Table 2**

**Correlation Matrix of Psychotic-Like Experiences.** *r_s_* = Spearman’s rho; CI = confidence interval; *p* = probability; SPQ = Schizotypal Personality Questionnaire; PAGE-R = revised Exceptional Experiences Questionnaire. The critical thresholds provided by the FDR procedure (Benjamini & Hochberg, 1995) were .004 (*α* = .10, trend), .002 (*α* = .05, significant), and .000 (*α* = .01, highly significant). Significant correlations are in boldface, statistical trends in italics.

|  |  | |  |  | | |
| --- | --- | --- | --- | --- | --- | --- |
|  | *r_s_* [CI 95%], *p* | | | | |  |
|  |  |  | | |  |  |
|  | SPQ ideas of reference | SPQ paranormal beliefs | | | SPQ unusual perceptual experiences |  |
| SPQ Paranormal beliefs | **.40 [.28, .51], .000** |  | | |  |  |
| SPQ Unusual perceptual experiences | **.46 [.34, .59], .000** | **.55 [.45, .64], .000** | | |  |  |
| SPQ Suspiciousness | **.46 [.34, .59], .000** | **.19 [.05, .32], .006** | | | **.34 [.22, .46], .000** |  |
| PAGE-R Odd beliefs | **.50 [.39, .59], .000** | **.69 [.61, .76], .000** | | | **.66 [.57, .73], .000** |  |
| PAGE-R Dissociative anomalous perceptions | **.43 [.31, .53], .000** | **.48 [.36, .57], .000** | | | **.54 [.44, .63], .000** |  |
| PAGE-R Hallucinatory anomalous perceptions | **.35 [.22, .46], .000** | **.48 [.37, .58], .000** | | | **.55 [.45, .64], .000** |  |
|  |  |  | | |  |  |
|  | SPQ suspiciousness | PAGE-R odd beliefs | | | PAGE-R Dissociative anomalous perceptions |  |
| SPQ Paranormal beliefs |  |  | | |  |  |
| SPQ Unusual perceptual experiences |  |  | | |  |  |
| SPQ Suspiciousness |  |  | | |  |  |
| PAGE-R Odd beliefs | **.36 [.23, .47], .000** |  | | |  |  |
| PAGE-R Dissociative anomalous perceptions | **.31 [.18, .43], .000** | **.61 [.52, .69], .000** | | |  |  |
| PAGE-R Hallucinatory anomalous perceptions | **.40 [.28, .51], .000** | **.63 [.54, .71], .000** | | | **.60 [.50, .68], .000** |  |
